# Insights into chestnut (*Castanea* spp.) graft incompatibility through the monitoring of chemical and physiological parameters

**DOI:** 10.1007/s00425-025-04639-8

**Published:** 2025-02-14

**Authors:** Giovanni Gamba, Dario Donno, Burak Akyüz, Beatriz Cuenca Valera, Gabriele Loris Beccaro

**Affiliations:** 1 DiSAFA, Dipartimento Di Scienze Agrarie, Forestali E Alimentari, Università Di Torino, 10095 Grugliasco TO, Italy; 2 Centro Regionale Di Castanicoltura del Piemonte, 12013 Chiusa Di Pesio CN, Italy; 3 Faculty of Agriculture, Department of Horticulture, Ondokuz Mayıs University, 55139 Samsun, Türkiye; 4 Empresa de Transformación Agraria S.A., S.M.E., M.P. (TRAGSA), 32700 Maceda, OU Spain

**Keywords:** Chlorophyll content, Clonal rootstocks, Green extraction, Phenolic compounds, Predictive markers, SPAD, Stomatal conductance, Tannins

## Abstract

**Main conclusion:**

Incompatible chestnut grafts exhibited a notably reduced stomatal conductance, mirroring the trend observed for leaf chlorophyll content. Woody tissues at the graft interface of these combinations showed a significantly higher total phenolic content, especially in the internal layers.

**Abstract:**

In recent years, significant efforts have been made to study the mechanisms of graft incompatibility in horticultural species, though research on minor species like chestnut remains limited. This study investigated the physiological and chemical dynamics in various chestnut grafts, aiming to develop a method for the early detection of graft incompatibility. The total phenolic content (TPC) and specific phenolic markers were analyzed at two phenological stages, callusing (CAL) and end of the vegetative cycle (EVC), using spectrophotometric and chromatographic techniques. These analyses were performed on three sections comprising the graft. Stomatal conductance (G_sw_) and leaf chlorophyll content were assessed during the growing season as support tools, being non-destructive useful indicators of plant water status. Significant differences in the physiological traits among compatible and incompatible grafting combinations were evident and remained stable throughout the season. Compatible combinations consistently displayed greater leaf chlorophyll content and higher stomatal conductance, highlighting their superior physiological performance. TPC increased significantly from the CAL to EVC stage across all experimental grafting combinations and in all three analyzed sections. Greater phenol accumulation was observed at the graft union of incompatible combinations, particularly in the inner woody tissues. The phytochemical fingerprint revealed castalagin as the dominant compound, with significant increases in benzoic acids, catechins, and tannins during the growing season. However, the role of gallic acid and catechin as markers of graft incompatibility remains uncertain. The multidisciplinary approach provided valuable insights into the issue of graft incompatibility.

**Graphical abstract:**

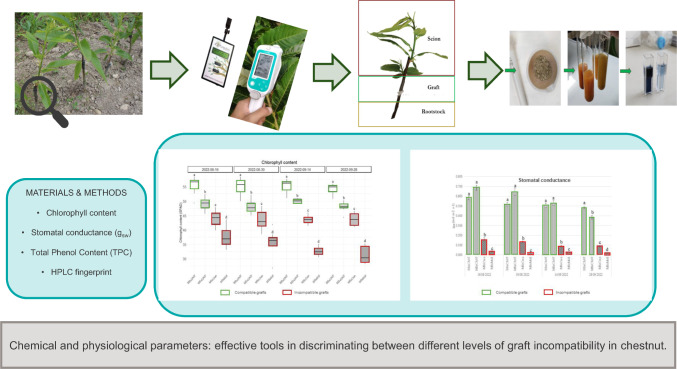

**Supplementary Information:**

The online version contains supplementary material available at 10.1007/s00425-025-04639-8.

## Introduction

In nature, grafting is a phenomenon in which two plant segments connect and grow as a single entity, thanks to the formation of a vascular continuity between them. It is reasonable to think that artificial grafting was first performed observing the natural process, as many techniques known as “approach grafting” took similar forms (Garner 2013). Historical sources testify how this technique has been practiced for millennia on perennial crops by ancient Chinese and Greeks (Melnyk and Meyerowitz 2015). In the last century, grafting took hold also on horticultural species, mainly belonging to the Solanaceae and Cucurbitaceae families (Goldschmidt 2014).

The importance of grafting using selected genotypes as rootstocks is well recognized. Grafting significantly impacts crop production and quality, influencing many morpho-physiologic traits such as canopy architecture, nutritional uptake, vigor, tolerance or resistance to pests and diseases, flowering time, cold hardiness, and resistance to replant disease (Nimbolkar et al. 2016; Dogra et al. 2018). Rootstocks can impart resistance or increased tolerance to abiotic stresses like salinity, hypoxia, toxicity of heavy metals, and stress connected to soil pH, which are becoming more frequent as a consequence of climate change (Schwarz et al. 2010; Baron et al. 2019).

Successful grafts pass through a series of morphological, physiological, and molecular events consisting in hormonal signaling, protein turnover, gene expression, phenol metabolism, and ion uptake and transport ending with the establishment of the vascular connection. Incompatible grafts result from the failure of one or more of these events (Adhikari et al. 2022). Disaffinity among two genotypes can be due to various factors: taxonomic distance, pathogens, environmental conditions, and poor craftsmanship above all (Loupit and Cookson 2020).

Over the past decades, considerable efforts have been made to study the mechanisms underlying grafting formation, with the aim of understanding the causes of graft incompatibility and identifying potential metabolic markers to be employed in breeding programs. Research has been focusing on the principal cash crops, while few works are available in literature for minor species.

Many studies focused on the accumulation of phenolic compounds at the graft union, as these secondary metabolites play a primary role in many metabolic processes such as cell division, development, and differentiation (Loupit and Cookson 2020). Moreover, being involved in defense responses, their increased biosynthesis could be a mechanism with which plants try to hinder the oxidative stress related to graft incompatibility (Pereira et al. 2017). However, the concentration of certain phenolic molecules could limit the proliferation and differentiation of callus, thereby hindering the formation of new vascular tissues (Prabpree et al. 2018). Qualitative and quantitative analysis of phenol concentration has highlighted the role of certain molecules as promising markers of graft incompatibility on several species, especially with new cultivar/rootstock combinations (Musacchi et al. 2000; Zarrouk et al. 2010; Azimi et al. 2016).

Beside chemical traits, rootstocks may considerably affect many physiological processes occurring in the upper part of the grafted trees, namely the scions. Effects on the physiology could be linked to the root system, especially in the case of dwarfed trees, but also to the graft incompatibility among genotypes. According to the grafting combination, rootstocks can interfere with stomatal opening and closure, net CO_2_ assimilation, and intracellular CO_2_ through hydraulic and hormonal signaling (Gonçalves et al. 2006; Adhikari et al. 2022). Stomatal opening is usually expressed as stomatal conductance (g_s_), a useful parameter evaluating the rate of water vapor exiting or CO_2_ entering through stomata, which is a measure of leaf transpiration. As the incomplete wound healing in incompatible unions causes vascular discontinuity affecting, therefore, the plant water status, these physiological parameters could help in the early diagnosis of incompatibility before visual symptoms occur (Corelli-Grapadelli et al. 2000; Losciale et al. 2008; Gamba et al. 2023).

At the early stage of grafting process, incompatible combinations may show a complete or partial vascular discontinuity associated with phloem degeneration at the graft union. This condition could affect the activity of the new phloem and xylem, leading to detrimental effects on the ascendent water flow and on the descendent photo-assimilates (Pina and Errea 2005). Beside gas exchange measurements, analysis of the leaf chlorophyll content proved to be an efficient tool to discriminate among different degrees of compatibility (Tedesco et al. 2020). A practical way to assess this parameter is via a handheld SPAD meter, a device widely used for the rapid and non-destructive assessment of the chlorophyll content. It gives relative SPAD values proportional to the amount of leaf chlorophyll. Many studies on different horticultural species confirmed the effectiveness of this parameter in corroborating analytical evidence of graft incompatibility (Chen et al. 2016; das Neves et al. 2017; He et al. 2022; Wang et al. 2022).

At present, few research works are available on chestnut graft incompatibility. Beside the historical, ecological, and cultural relevance that this species has represented for centuries, it must be remembered that chestnut trees have represented and still represent a considerable economic resource (fruit, wood, tannin production, and honey) for mountainous and hilly areas (Beccaro et al. 2020). Despite having gone through a gradual decline during the twentieth century, in the last decades, the interest for its cultivation has been renewed. Intensive orchards are rising also in lowland areas, adopting agricultural practices typical of major fruit crops. Among them, the use of clonal rootstocks is of great interest. Consequently, breeding activity is enhancing, and new resilient genotypes are presently being released. Graft incompatibility is, therefore, a topical issue that limits the adoption of new rootstocks and cultivars, crucial materials for the revitalization of the chestnut cultivation (Vahdati et al. 2021).

The present study explored the physiological and chemical dynamics at the graft union, above and below it in different intraspecific and interspecific chestnut grafts. In particular, the implications of phenolic compounds during grafting development of compatible and incompatible combinations were reviewed at two phenological times. Stomatal conductance and leaf chlorophyll content were assessed during the growing season as support tools, being non-destructive useful indicators of plant water status. The aim of the present study was to identify an effective approach for an early detection of incompatibility in chestnut propagation, to accelerate rootstock breeding and the development of resilient materials.

## Materials and methods

### Plant material

**Fig. 1:**
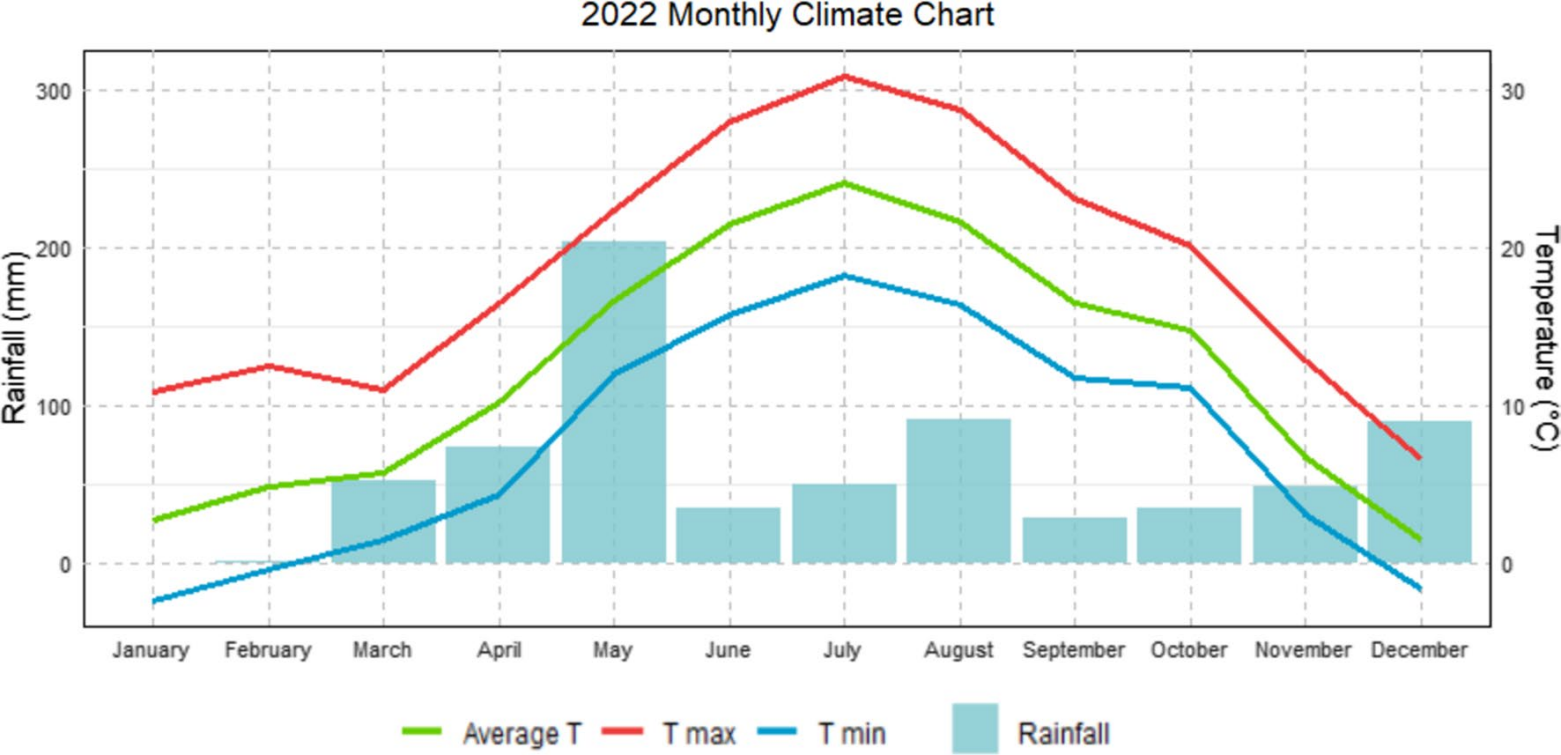
Climate graph representing the average monthly rainfall and temperatures (2022) in Chiusa di Pesio, Cuneo province (source: Arpa Piemonte)

Plant materials consisting in grafted trees were grown at the Chestnut R&D Center Piemonte in Chiusa Pesio (44°18′ 21′′ N 7°40′ 50′′ E, 620 m above mean sea level). Figure 1 shows the monthly rainfall and temperatures recorded at the experimental site in 2022.

Scions were collected in February 2022 from the *Castanetum* germplasm collection field located at the Chestnut R&D Center and dark-stored at 4 °C until grafting. The mother plants were certified in terms of genotype trueness-to-type and phytosanitary requirements to avoid the influence of diseases during graft development. Grafts were performed in March 2022 using the whip and tongue technique, which ensures high success rate and good stability thanks to the tongue that holds rootstock and scion together. The trial consisted in the use of 2-year-old clonal rootstocks cultivar “Marsol CA07” which was self-produced via cutting at the Chestnut R&D Center and two seedling rootstocks of *Castanea mollissima* and *C. crenata* grown in the Castanetum. Four experimental combinations were selected based on their compatibility level (Table 1) and grown in open field.

**Table 1:** Experimental grafting combinations chosen for the study, with their known compatibility level

Code	Rootstock	Scion	Compatibility level
BBxCA07	Marsol CA07 (*C. crenata x C. sativa*, clonal)	Bouche de Bétizac (*C. crenata x C. sativa*)	Compatible
MSxCA07	Marsol CA07 (*C. crenata x C. sativa*, clonal)	Marrone Val Susa (*C. sativa*)	Compatible
MSxCren	*C. crenata* (seedling)	Marrone Val Susa (*C. sativa*)	Incompatible
MSxMoll	*C. mollissima* (seedling)	Marrone Val Susa (*C. sativa*)	Incompatible

The reference combinations for a positive degree of compatibility were selected based on their well-documented affinity. Marsol CA07 is the predominant rootstock in modern orchards due to its high genetic compatibility with major French cultivars (Bounous et al. 1997; Bounous 2002), particularly Bouche de Bétizac (*C. sativa* × *C. crenata*), as well as with most *C. sativa* cultivars (Beccaro et al. 2019). Many chestnut orchards established over 30 years ago with Bouche de Bétizac and Marrone-type cultivars grafted onto Marsol CA07 remain productive today, showing no signs of incompatibility.

This strong compatibility is supported by their phylogenetic proximity. The genotypes composing the Bouche del Bétizac × Marsol CA07 combination [(*C. sativa* × *C. crenata*) × (*C. crenata* × *C. sativa*)] belong to the same species, while the genotypes of the Marrone Val Susa × Marsol CA07 combination [*C. sativa* × (*C. crenata* × *C. sativa*)] are also closely related.

Graft samples for the chemical analysis were collected at two phenological stages: callusing (CAL) and end of vegetative cycle (EVC), in line with previous studies on chestnut and other major fruit species (Canas et al. 2015; Gamba et al. 2021 and 2023). The first sampling time corresponds to approximately 60 days after grafting (DAG), while EVC takes place during the dormancy period, at the end of the year. Experimental graft combinations were cut 5 cm above and below graft union, to include scion and rootstock tissues. Ungrafted plant materials were also sampled, so as to have information about the content in phenol compounds in the starting genotypes. Samples were then stored at − 80 °C until further analysis on woody tissues.

### Sample preparation

The extraction of secondary metabolites was carried out on external and internal tissues, independently. Bark, cambium, and phloem were separated from the inner tissues; afterwards, they were ground to powder in a mortar. Liquid nitrogen was used to facilitate grinding operations and to maintain the cold chain, essential to limit phenol degradation. Finally, milled tissues were weighed and stored at − 80 °C until further analysis (Donno et al. 2019; Gamba et al. 2021).

### Extraction of secondary metabolites from woody tissues

For the extraction of phenol compounds, a blended approach was employed, consisting in a conventional extraction method via a solvent followed by an ultrasound-assisted extraction. 1 g of sample was placed into 20 mL of a mix of methanol, water, and HCl (95:4.5:0.5, by vol) overnight. Then tubes containing sample and extraction solvent were moved into an ultrasonic bath for 30 min at 23 kHz (Reus sarl, Drap, France). Later, each sample was centrifuged at 3600 g for 10 min and finally stored at 4 °C and 95% relative humidity until further analysis.

### Total phenolic content (TPC) assessment

The total phenolic content was assessed using the Folin-Ciocalteu method (Slinkard and Singleton 1977), which was partially modified (Donno et al. 2022). Absorbance was read with a 1600-PC single-beam UV–Vis spectrophotometer (VWR International, Milan, Italy) at a wavelength of 760 nm. Results were expressed in mg of gallic acid equivalents (GAE) per 100 g of fresh weight (FW).

### Bioactive compounds characterization

The following phenolic classes have been considered for the phytochemical fingerprint, according to previous studies (Cooman et al. 1996; Hudina et al. 2014; Canas et al. 2015; Gamba et al. 2021): catechins (catechin, epicatechin), benzoic acids (ellagic and gallic acids), and tannins (castalagin, vescalagin). The liquid phase of the samples previously prepared was filtered through a 0.45-μm filter (polytetrafluoroethylene membrane—PTFE) and finally analyzed by HPLC–DAD (Donno et al. 2021). For the analysis, an Agilent 1200 high-performance liquid chromatograph–UV–Vis diode array detector (Agilent Technologies, Santa Clara, CA, USA) was used. The molecules were separated on a Kinetex C18 column (Phenomenex, Torrance, CA, USA) (Fioccardi et al. 2022). The chromatographic conditions of the method used for the characterization of the benzoic acids, catechins, and tannins are reported in Table 2.

**Table 2:** Chromatographic conditions of the method used for the identification and quantification of benzoic acids, catechins, and tannins

Class of interest	Stationary phase	Mobile phase	Wavelenght
Benzioc acids, catechins and tannins	KINETEX—C18 column (4.6 × 150 mm, 5 μm)	A: H_2_O/CH_3_OH/HCOOH (5:95:0.1 v/v/v), pH = 2.5; B: CH_3_OH/HCOOH (100:0.1 v/v)	280

Elution conditions—gradient analysis: 3%B to 85%B in 22 min + 85%B in 1 min (2 min conditioning time); flow: 0.6 mL min^−1^

### Gas exchanges and chlorophyll assessment

Physiological responses of chestnut to graft incompatibility were assessed measuring the chlorophyll content and the stomatal conductance to water (g_sw_). The first parameter was recorded with Arborcheck^®^ ArbCm 01 (Hansatech, Pentney, UK) and expressed as SPAD units, which are estimates of chlorophyll content of the leaf. A steady-state handheld LI-600 porometer (LI-COR Environmental, Lincoln, NE, USA) was used to measure leaf-level stomatal conductance, expressed in mol m^−2^ s^−1^. In particular, LI-600 evaluates stomatal conductance to water vapor (g_sw_), which gives a rate of water vapor exiting through stomata. Measurements were taken every 15 days from August to September, from 11:00 to 13:00. Monitoring was carried out on 30 fully developed sun-facing leaves belonging to 5 trees per combination, choosing days with comparable environmental conditions. Leaves were randomly selected from the shoots located all around the crown. For each monitoring day, both parameters were evaluated on the same leaves, avoiding midrib or any other large vein, chlorotic areas, and holes. Instruments were placed in similar positions in the bottom-right section, choosing a suitable point mid-way between the outer edge of the leaf and the midrib (Zarrouk et al. 2006; Gamba et al. 2023).

### Statistical analysis

Chemical and physiological results were evaluated through analysis of variance (ANOVA) for comparison of means (R Studio, version 2022.02.2 + 485). Significant statistical differences among biological repetitions were investigated using the Tukey’s HSD multiple range test (*P* < 0.05). Where normality or homoscedasticity assumptions were not met, the Kruskal–Wallis analysis of variance was applied, assessing any significant differences with Duncan’s multiple range test (*P* < 0.05).

## Results

### Gas exchanges and chlorophyll assessment

#### Leaf chlorophyll content

**Fig. 2:**
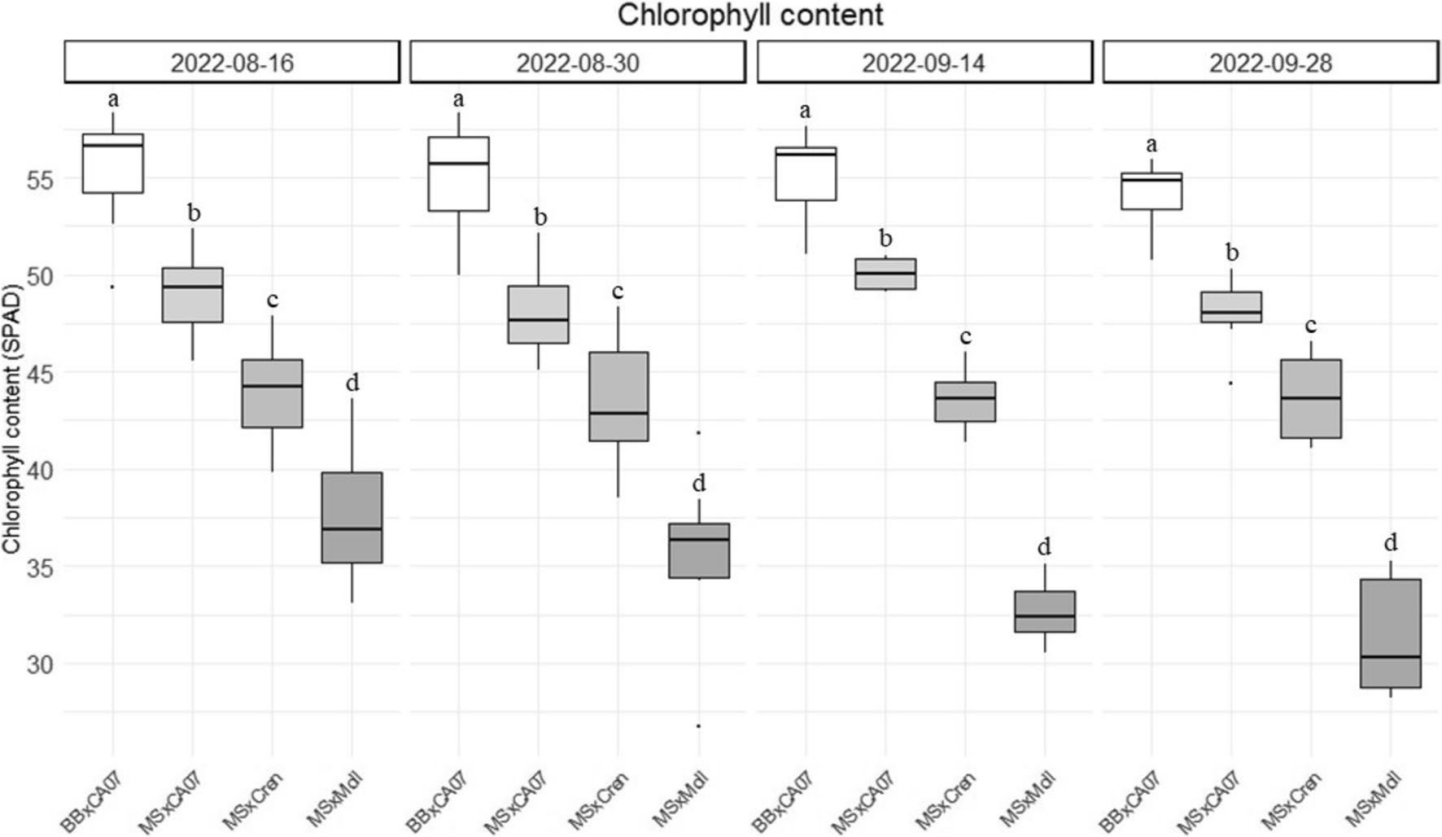
Leaf chlorophyll content of the experimental graft combinations measured during the vegetative cycle and expressed in SPAD units. Bouche de Bétizac x Marsol CA07 (BBxCA07), Marrone Val Susa x Marsol CA07 (MSxCA07), Marrone Val Susa x *C. crenata* (MSxCren), Marrone Val Susa x *C. mollissima* (MSxMoll). Mean value and standard deviation are given for each sample (*n* = 30). Different letters for all the considered groups indicate significant statistical differences (*P* < 0.05)

The measurements on the chlorophyll content revealed statistically significant differences across all experimental combinations, which remained consistent throughout the vegetative cycle, as illustrated in Fig. 2. In particular, the BBxCA07 combination exhibited the highest average leaf chlorophyll content, reaching 55.50 ± 2.78. This was followed by the MSxCA07 combination, with an average content of 48.84 ± 1.68. Among the incompatible combinations, MSxCren recorded the intermediate value of 43.64 ± 2.28, while MSxMoll had the lowest content of chlorophyll, averaging 34.28 ± 2.97.

During the growing season, the SPAD index had a physiological decrease, to a greater extent in the case of MSxMoll (− 16.53%). The compatible combinations had a similar downward trend (− 2.27% for BBxCA07 and − 2.28% for MSxCA07), while MSxCren had the lowest reduction (− 0.68%).

**Fig. 3:**
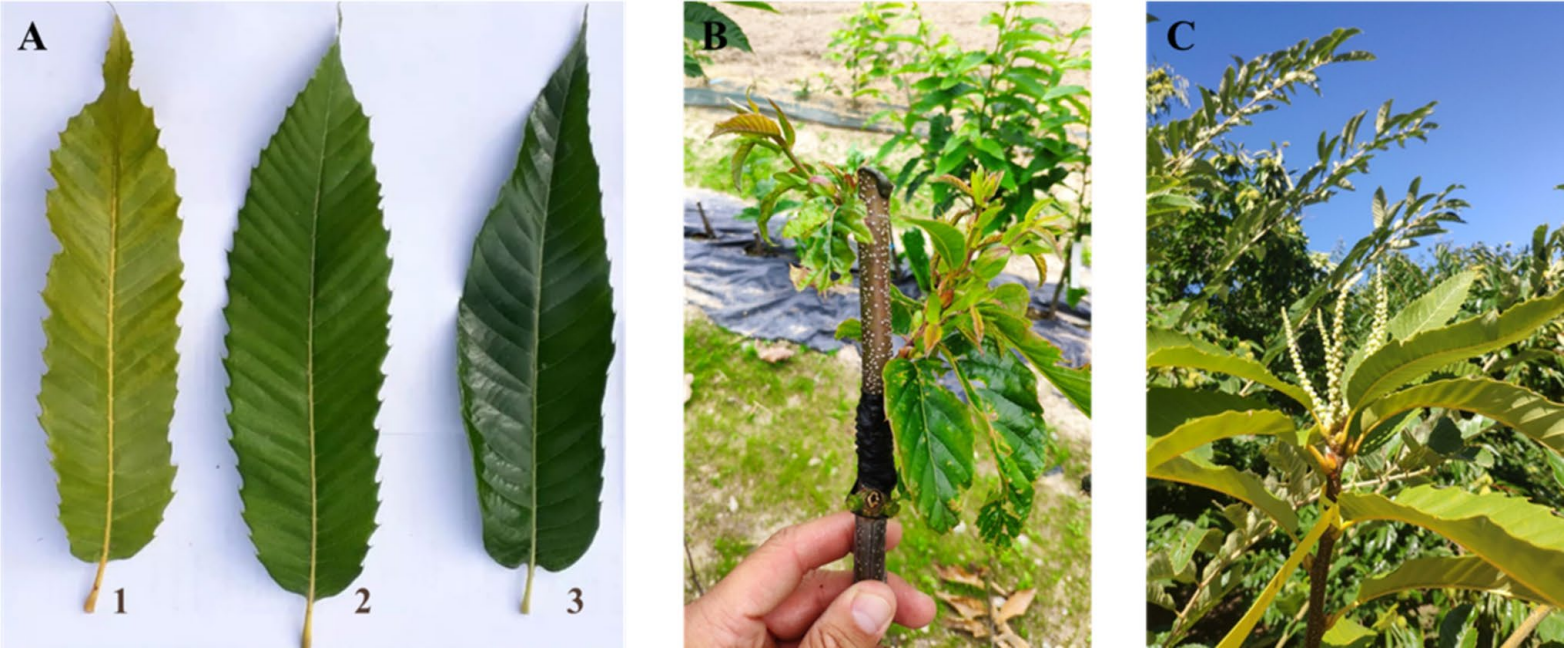
**A** Visible difference in the chlorophyll content measured on September 14, 2022 (leaf 1. Marrone Val Susa x *C. mollissima* - MsxMoll; 2. Marrone Val Susa x *C. crenata* - MSxCren; 3. Bouche de Bétizac x Marsol CA07 - BBxCA07). **B**, **C** Typical symptoms of graft incompatibility: yellowing and curling of the leaves and unhealthy appearance of shoots (**B**) and early flowering (**C**), as reported by Vahdati et al. 2021

The observed differences in leaf chlorophyll content suggest varying levels of graft compatibility. The highest values were observed in the BBxCA07, which is the genetically closest combination [(*C. sativa* x *C. crenata*) x (*C. crenata* x *C. sativa*)]. Conversely, the lowest values were recorded in the MSxMoll combination (*C. sativa* x *C. mollissima*), the most genetically distant. Figure 3 reports typical symptoms of graft incompatibility observed during the experiment.

#### Stomatal conductance

**Fig. 4:**
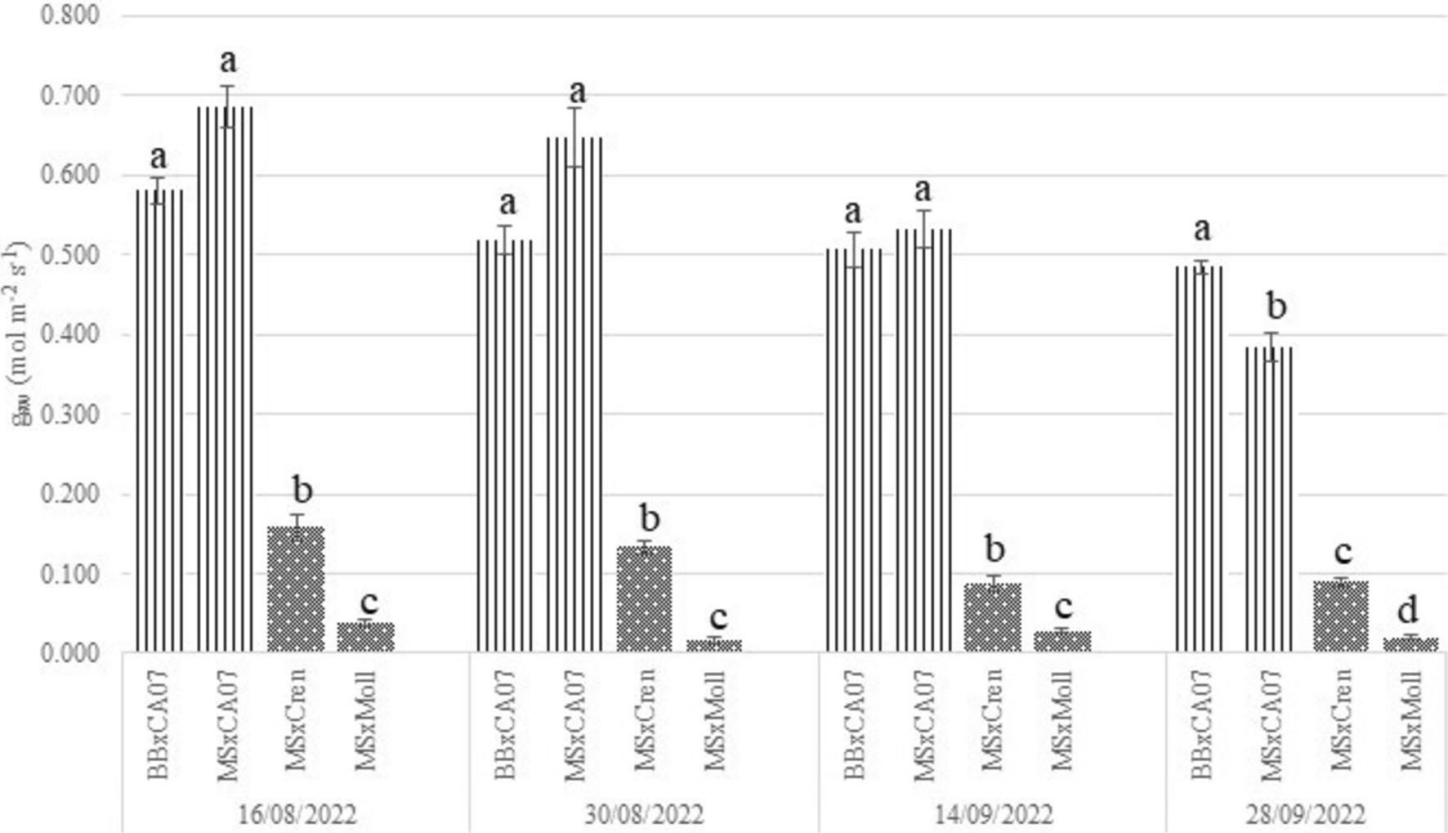
Stomatal conductance to water vapor (g_sw_) for the experimental grafting combinations, expressed as mol m^−2^s^−1^. Bouche de Bétizac x Marsol CA07 (BBxCA07), Marrone Val Susa x Marsol CA07 (MSxCA07), Marrone Val Susa x C. crenata (MSxCren), Marrone Val Susa x *C. mollissima* (MSxMoll). Means (*n* = 30) followed by the same letter are not significantly different at *P* < 0.05 (Duncan’s test)

Stomatal conductance was assessed via porometer on the same leaves used for the measurement of the chlorophyll content. The results of stomatal conductance recorded during the vegetative season, expressed as the rate of water vapor exiting through stomata, are reported in Fig. 4.

Significant differences can be clearly observed among compatible (striped bars) and incompatible (dotted bars) grafting combinations. BBxCA07 and MSxCA07 performed similarly, with average values of 0.526 ± 0.046 mol m^−2^ s^−1^ and 0.571 ± 0.066 mol m^−2^ s^−1^, respectively. MSxCren differed from both the above-mentioned compatible combinations and from MSxMoll, with an average value of 0.115 ± 0.025 mol m^−2^ s^−1^. Finally, MSxMoll recorded the lowest average value of 0.026 ± 0.010 mol m^−2^ s^−1^.

Differences remained consistent throughout the growing season, with greater values for Bouche de Bétizac and Marrone Val Susa grafted onto clonal hybrid rootstock Marsol CA07. Only the last measurement highlighted differences among these two combinations, with a higher average value for BBxCA07. The pattern that emerged from the monitoring of stomatal conductance to water (g_sw_) is similar to the one observed with the leaf chlorophyll content. For both the parameters, the incompatible combinations performed the lowest values, in particularly in the case of MSxMoll.

### Chemical dynamics

#### Total phenolic content

The TPC was measured in the inner and outer tissues of the three sections composing the graft. Table 3 reports the values obtained for each section and in each woody layer of the experimental grafting combinations tested.

**Table 3:** Total phenolic content measured in the three sections composing the grafts and in the inner and outer woody layers

Sampling time	Section	Woody layer	BBxCA07		MSxCA07		MSxCren		MSxMoll	
			Compatible		Compatible		Incompatible		Incompatible	
			Mean	SD	Mean	SD	Mean	SD	Mean	SD
			(Mg GAE/100 g FW)		(Mg GAE/100 g FW)		(Mg GAE/100 g FW)		(Mg GAE/100 g FW)	
Callusing (CAL)	Scion	External	2923.44 b	21.04	3109.00 a	61.10	3138.25 a	45.59	3182.84 a	8.53
		Internal	391.63 b	2.81	303.17 c	13.40	463.25 a	9.42	485.72 a	15.73
	Graft union	External	2800.90 a	20.25	2813.47 a	21.70	2863.00 a	61.22	2212.07 b	85.39
		Internal	529.55 b	22.22	403.17 c	19.16	739.30 a	33.40	669.19 a	51.16
	Rootstock	External	2892.50 c	79.49	3028.88 bc	15.62	3186.45 a	28.87	3036.59 b	63.46
		Internal	291.65 b	21.35	270.86 c	11.44	364.50 a	14.90	432.31 a	20.44
End of vegetative cycle (EVC)	Scion	External	3168.34 c	38.82	3307.99 a	12.64	3208.11bc	7.22	3276.50 ab	38.71
		Internal	593.39 d	12.29	1232.25 c	27.41	1547.67 b	97.80	1749.89 a	23.47
	Graft union	External	3283.67 ab	5.21	3329.86 a	80.90	3210.60 b	20.42	3236.21 ab	16.74
		Internal	791.26 c	9.12	1293.67 b	17.52	3110.13 a	24.18	3149.67 a	5.97
	Rootstock	External	3166.30 b	6.73	3244.98 a	29.51	3215.55 ab	14.40	2959.03 c	29.81
		Internal	465.43 d	7.27	814.21 b	3.64	694.70 c	36.17	945.22 a	27.60

*BBxCA*07 Bouche de Bétizac x Marsol CA07, *MSxCA*07 Marrone Val Susa x Marsol CA07, *MSxCren* Marrone Val Susa x *C. crenata*, *MSxMoll* Marrone Val Susa x *C. mollissima*. The mean value and standard deviation are given for each sample (*n* = 3). Different letters for each compound indicate the significant differences at *P* < 0.05

**Fig. 5:**
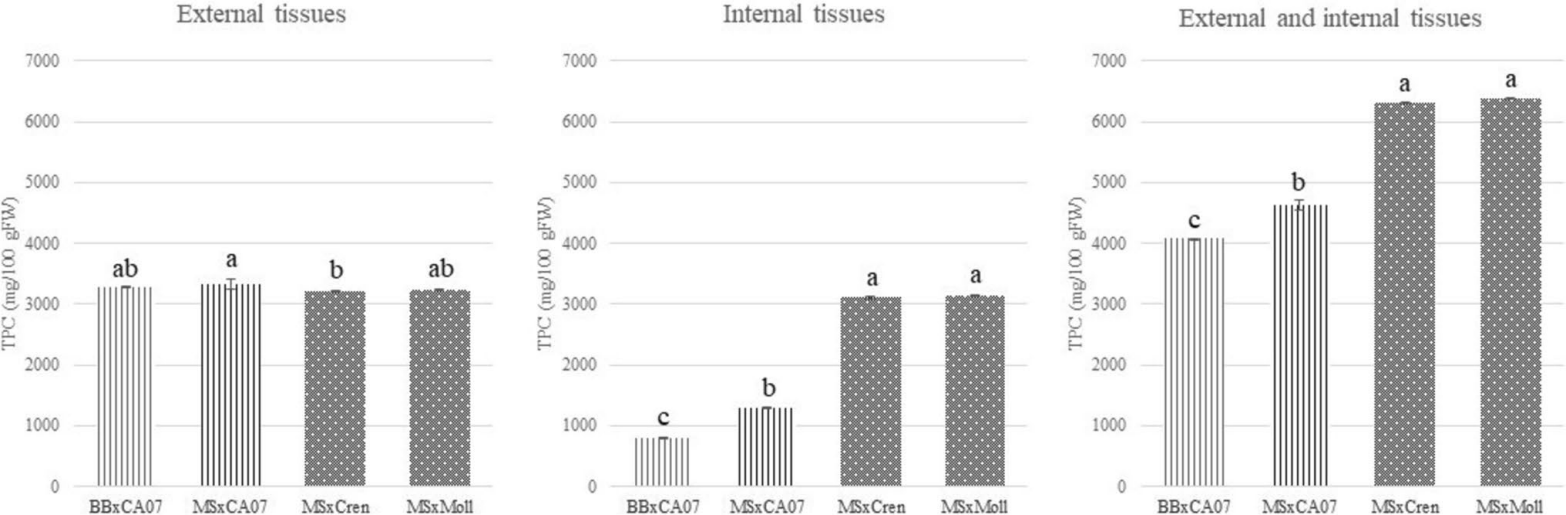
Total phenolic content in the external and internal woody layers of graft union, values measured at the EVC stage (end of the vegetative cycle). Bouche de Bétizac x Marsol CA07 (BBxCA07), Marrone Val Susa x Marsol CA07 (MSxCA07), Marrone Val Susa x *C. crenata* (MSxCren), Marrone Val Susa x *C. mollissima* (MSxMoll). The mean value and standard deviation are given for each sample (*n* = 3). Different letters for each compound indicate the significant differences at *P* < 0.05

TPC was quantified also on the inner and outer tissues of the ungrafted genotypes, namely on Bouche de Bétizac, Marrone Val Susa, Marsol CA07, *C. mollissima*, *C. crenata*. Knowing the initial quantities of phenol compounds in each genotype was useful for the interpretation of the results. The amount of TPC increased statistically from CAL to EVC in all the sections of all the combinations, as expected, except for the outer tissues of scion and rootstock of MSxCren and MSxMoll. The external tissues composed of bark, cambium, and phloem, had a significant higher content of phenol compounds compared to the internal ones for all the sections analyzed (scion, graft union, and rootstock). These differences remained consistent during the growing season though no patterns emerged by analyzing the TPC in the external layers. A rather different behavior was observed in the inner tissues, where the amount of phenol compounds turned out to be higher in the incompatible combinations for all the sections. In particular, the amount of TPC at the graft union was over three times higher at EVC in the case of incompatible combinations (Fig. 5).

As shown in Fig. 5, a greater accumulation of phenolic compounds was observed in the graft area of the incompatible unions MSxCren and MSxMoll (dotted bars), especially in the inner woody layers, with an average of 3129.90 ± 15.07 mg GAE/100 g FW. Between BBxCA07 and MSxCA07 (compatible combinations, striped bars), the latter recorded a statistically higher amount of TPC (1293.67 vs 791.26 mg GAE/100 g FW). These small differences among compatible unions could suggest, as observed with the leaf chlorophyll content, varying levels of graft compatibility.

At CAL, the content of phenol compounds, especially in the external tissues, seemed to be influenced primarily by the physiological enhancement of phenol metabolism and by the genotype. In contrast, at EVC, differences in phenol accumulation were significantly more pronounced, especially at the graft union and in the internal tissues. These differences seem to be related to the incompatibility between the tested genotypes, in accordance with previous scientific studies on other species.

#### Bioactive compounds identification and quantification

The study focused on markers belonging to three phenolic classes: catechins (catechin, epicatechin), benzoic acids (ellagic and gallic acids), and tannins (castalagin, vescalagin). All these compounds exhibited a significant increase during the growing season, reaching their highest levels at the EVC stage (Table S1 and S2). This trend aligns with the previously presented TPC results, which demonstrate a progressive polyphenolic biosynthesis over the vegetative period, particularly in incompatible unions.

**Fig. 6:**
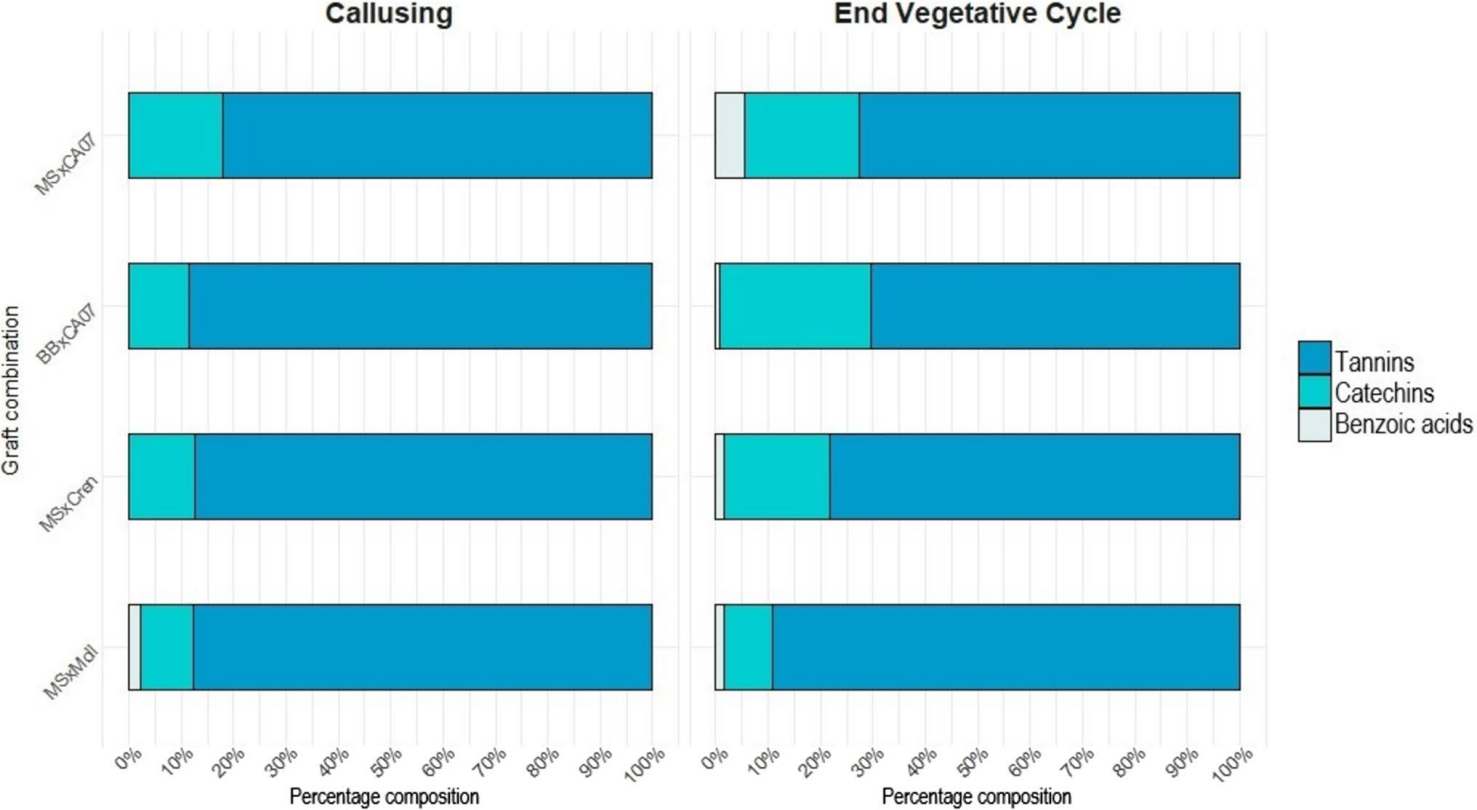
Phytochemical fingerprint of the woody tissues comprising the graft union (results combine data from both outer and inner tissues). Bouche de Bétizac x Marsol CA07 (BBxCA07), Marrone Val Susa x Marsol CA07 (MSxCA07), Marrone Val Susa x *C. crenata* (MSxCren), Marrone Val Susa x *C. mollissima* (MSxMoll)

Figure 6 illustrates the percentage composition of the three classes considered in the woody tissues (association of outer and inner) at the graft union. Results primarily focus on the graft union, as this section, as observed in other fruit species, would likely be the site where polyphenol biosynthesis is most influenced by the incompatibility phenomenon. Tannins represented the predominant phenolic class, consistently exceeding 70% across all combinations, with a maximum concentration of 89.05% in MSxMoll. Castalagin was highlighted as the predominant compound within this class. The analysis of the ungrafted starting materials revealed that tannin content was strongly influenced by genotype. In particular, tissues of *C. sativa* (5002.26 ± 101.48 mg/100 g_FW_) showed nearly twice the tannin concentration compared to Asiatic species (*C. crenata,* 2634.61 ± 63.93 mg/100 g_FW_ and *C. mollissima,* 2556.36 ± 140.75 mg/100 g_FW_).

Variations in benzoic acids content were particularly evident throughout the season, potentially linked to enhanced biosynthesis induced by the grafting process. However, their proportion remained low compared to other phenolic classes, peaking at 5.36% in MSxCA07 and dropping to 0.59% in BBxCA07.

Benzoic acids, similar to catechins, did not exhibit univocal trend across the experimental combinations. Gallic acid increased throughout the growing season, with higher levels observed in the compatible combination MSxCA07 (160.34 ± 7.23 mg/100 g_FW_). The initial concentration of gallic acid in the starting materials was undetectable, whereas ellagic acid was present in very low quantities only in the outer tissues of Marsol CA07 and in the Asian species (*C. mollissima*, 8.25 ± 1.93 mg/100 g_FW_ and *C. crenata*, 9.85 ± 0.36 mg/100 g_FW_). Similarly, catechin was undetected in the woody tissues of the starting materials, which, however, were particularly rich in epicatechin, especially in the outer layers. Furthermore, catechin was present in substantial amounts in the compatible graft unions, especially in BBxCA07 (545.04 ± 31.78 mg/100 g_FW_). These findings represent a future challenge in the evaluation of their effectiveness as markers of graft incompatibility in chestnut.

## Discussion

To date, few studies have focused on the physiological parameters of chestnut, and even fewer have explored their potential as early predictive markers of graft incompatibility. Camisón et al. (2020) conducted hydric stress experiments, including water deficiency and waterlogging, using 2-year-old European chestnut (*Castanea sativa*) seedlings. Among other parameters, they studied the chlorophyll content by measuring the SPAD index. Control trees subjected to a well-watered regime exhibited slightly lower values compared to those observed in the compatible graft combinations in the present study. Another research done by Dinis et al. (2011) focused on *Castanea* spp. plantlets inoculated with *Phytophthora cinnamomi* to better understand the mechanisms of chestnut resistance to ink disease (Dinis et al. 2011). Authors observed an opposite trend in total chlorophyll contents assessed via SPAD reading in the two clones under study related to pathogen infection. As for the study of Camisón et al. (2020), values are lower than the ones recorded in the grafting combination of the present study. This can be attributed to the differences in plant material under investigation (plantlets vs. grafted trees), which display varying levels of vigor, as well as to the contrasting growing conditions (pots vs. open field). However, the values obtained in the present work are supported by a preliminary study on chestnut graft incompatibility, where the same MSxCA07 combination exhibited similar SPAD index values (Gamba et al. 2023).

Moving on to other fruit *genera* such as *Citrus* or *Prunus*, numerous studies dealt with the leaf chlorophyll content related to graft incompatibility. A recent work investigated the changes in the leaf chlorophyll content of *Citrus maxima* cultivars grafted on different rootstocks (*Poncirus trifoliata* and *Citrus junos*). The incompatible combination exhibited the lowest chlorophyll content, with a severe decrease from 182 days after grafting (DAG) to 203 DAG (He et al. 2022). Another work on *Citrus* spp. by Wang et al. (2022) validates the use of SPAD reading as a pre-selection index for evaluating graft compatibility of stock and scion. Similar results were obtained on “Summergrand” nectarine cultivar (*P. persica*) grafted onto two different plum rootstocks, as reported by Amri et al. (2021). The comparison between graft combinations and ungrafted rootstocks highlighted lower chlorophyll content values in the case of the incompatible unions, accompanied by typical symptoms of the “translocated” incompatibility (yellowing and curling of the leaves, vigor reduction and unhealthy appearance of shoots, as shown in Fig. 3). A reduction in the rate of shoot growth due to graft incompatibility may lead to a decline in leaf carbon export from scion to rootstock, with consequently limited nitrogen assimilation (Moreno et al. 1994). As a result, chlorophyll content could drop due to the blockage of carbohydrate allocation (Zarrouk et al. 2006).

Another work on graft incompatibility in *Prunus* spp. evaluated the content of leaf chlorophyll of three peach cultivars grafted on different clonal rootstocks. Tree death caused by graft incompatibility was preceded by a drastic reduction in chlorophyll content values 5 months after field planting (das Neves et al. 2017). According to the findings of the present study, a previous experience (Gamba et al. 2023), and excluding any disorders due to pests, diseases, or nutrient deficiencies, chlorophyll contents lower than 40 SPAD units could, therefore, be indicators of stress related to graft incompatibility in chestnut, serving as a pre-selection index to support further chemical analyses as suggested for *Citrus* by Wang et al. (2022).

Similarly to chlorophyll content, stomatal conductance in *Castanea* spp. is poorly understood. Apart from the preliminary work by Gamba et al. (2023), whose findings are consistent with those of the present study, no other research explored the variations in stomatal conductance in relation to graft incompatibility in chestnut.

However, there are some studies on other fruit species that focused on the issue. Losciale et al. (2008) reports a study on pear ‘Bosc’ grafted on pear seedling and quince EMC, analyzing the effects of rootstocks on the photosynthetic efficiency under similar conditions of light and temperature (Losciale et al. 2008). Trees grafted on the incompatible rootstock EMC recorded lower stomatal conductance as a consequence of the limited hydraulic conductivity, which led to a reduced transpiration and net photosynthesis. These results confirm previous research on two pear cultivars grafted onto rootstocks with different degrees of graft compatibility; leaf transpiration, stomatal conductance, and net photosynthesis were reduced in the case of grafts with quince rootstock EM (Corelli-Grapadelli et al. 2000). Graft incompatibility among some pear cultivars and quince rootstock leads to a poor synthesis of vascular bundles at the graft union, inhibiting water and nutrient transport (Trinchera et al. 2013). Given the limited and often outdated research on this topic, much work remains to be done to correlate stomatal conductance with varying degrees of graft compatibility and to validate its potential as a pre-selection index for evaluating graft compatibility between rootstocks and scions across various fruit species.

Apart from physiological implications, as grafting represents a source of stress for plants, various chemical pathways are involved during scion-rootstock establishment. Among these, the metabolism of phenols is enhanced, as they are implicated in the processes of stress and wounding. These secondary metabolites play a primary role especially in the early growth stages of connections, being related to lignin formation and protein bounding (Errea 1998; Liu 2012).

Several studies reported an accumulation of TPC in the case of incompatible combinations, findings that align with the results of the present study (Rashedy and Hamed 2023; Jalali et al. 2024). It seems that the major chemical changes related to graft incompatibility occur at the graft union (Skočajić et al. 2021; Babar et al. 2023), as observed in the inner tissues of the experimental combinations under test, though examples of phenol translocation towards scion/rootstock have been reported. On chestnut, only Karadeniz et al. (1993) focused on the study of TPC dynamics during grafting establishment. In this preliminary experiment, different grafting techniques were compared at different times, assessing the success rate and the total phenolic content in phloem tissues of the graft union. Results showed that the amount of these secondary metabolites has been increasing all along the vegetative cycle, in accordance with the present findings. A recent study by Amri et al. (2021) revealed that incompatible peach/plum unions exhibited a statistically significant increase of TPC and PAL enzymatic activity in scion tissues at leaf fall period. Similarly, in the present study, a comparable accumulation was observed in the inner tissues of MSxCren and MSxMoll scions, suggesting a possible translocation of these secondary metabolites towards the upper part of the graft. Moreover, authors found a positive correlation between PAL activity and TPC, which leads them to believe that the change in the PAL activity might have a primary role in the accumulation of phenolic compounds (Amri et al. 2021). These data confirm an earlier chemical study on peach (*P. persica*) and Japanese apricot (*P. mume*) incompatible grafts (Pereira et al. 2017), where a translocation of these secondary metabolites in the scion occurred.

The assessment of the total phenolic content appears to be a valuable preliminary tool for distinguishing between compatible and incompatible combinations. However, a detailed identification and quantification of the key phenolic classes and specific molecules driving this accumulation could provide deeper insights into the mechanisms underlying graft incompatibility among genotypes.

The study examined several markers from the catechins, benzoic acids, and tannins classes, selected based on their reported role as potential indicators of graft incompatibility in the literature. All the compounds considered were mainly concentrated in the external woody tissues (Gamba et al. 2023) and their amounts increased along with the vegetative season, confirming the results of the TPC. Castalagin was the predominant compound found, as has already been observed on chestnut (Comandini et al. 2014; Gamba et al. 2021).

The levels of catechins and benzoic acids, particularly catechin and gallic acid—two compounds recognized as reliable markers of graft incompatibility in various fruit species, including *Pyrus*, *Vitis*, and *Castanea* (Hudina et al. 2014; Assunção et al. 2019; Gamba et al. 2021)—were higher at the graft interface of the compatible combinations tested. This observation aligns with a recent preliminary research on chestnut (Gamba et al. 2022), although some differences were observed in relation to *Pyrus* and *Vitis* spp.

Polyphenol compounds have been extensively investigated in many fruit species due to their proven role in graft formation, though studying the behavior of these biomolecules is challenging because of multiple reasons: influence of the environmental conditions, difficulties in finding homogenous nursery materials, establishment of fully incompatible combinations are among the main limitations. Regarding this latter aspect, experiences in the field seem to suggest that chestnut incompatibility may fall into the so-called localized incompatibility. Because of morpho-physiological alteration at the graft union, this type of disaffinity causes malformations which eventually leads to impaired union formation; after some years, grafted trees can break at the junction, with considerable economic losses (Beccaro et al. 2019; Rasool et al. 2020). Environmental stresses such as high sunlight exposure, extreme temperatures, pathogen infection, and nutrient or water deficiency can trigger secondary metabolic pathways like the phenylpropanoid pathway, responsible for the biosynthesis of metabolites such as flavonoids and tannins (Grace and Logan 2000). Field cultivation exposes plants to these variables, complicating the study of polyphenols. Finally, the genotype of the starting materials (scions and rootstocks) could also influence the phenolic levels at the graft interface, modifying the amounts of the selected incompatibility/compatibility markers. The aspects mentioned above render the study of graft incompatibility highly complex, thereby justifying the analysis of phenolic compounds in terms of classes rather than individual compounds in this work. Additional research is needed to establish concentration ranges, rather than precise values, to account for the variability associated with the aforementioned factors. Also, studying the dynamics of translocation could further clarify the role of certain polyphenolic markers in graft incompatibility.

While most of the studies focused on the assumption that the relationship between phenolic markers and graft incompatibility is linear, this may not necessarily be the case. Alternative correlation models may offer better explanations for data that sometimes appear incongruent when viewed solely through a linear lens, and future research on graft incompatibility must investigate potential non-linear correlations.

## Conclusion

This study investigated graft incompatibility in both intra- and inter-specific chestnut grafting using a multidisciplinary approach. Stomatal conductance and leaf chlorophyll content have proven to be effective and non-destructive validation tools. The monitoring of these physiological parameters during grafting formation allowed to clearly discriminate between compatible and incompatible grafting combinations.

The analysis of the total phenol content confirmed a pattern already observed in previous research on other fruit species. The biosynthesis of phenol compounds increased during the vegetative cycle and turned out to be higher at the graft union. At the end of the vegetative cycle, incompatible combinations showed the highest statistically significant values for TPC. The accumulation of polyphenols was concentrated in the inner tissues of the graft section. Finally, the phytochemical fingerprint indicated castalagin as the most represented compound. The biosynthesis of benzoic acids, catechins, and tannins increased significantly throughout the growing season, similarly to the increase observed in TPC. The role of gallic acid and catechin as markers of graft incompatibility remains uncertain, as their concentration was higher in the compatible grafts, contrary to expectations. Analysis of the individual markers did not reveal a clear trend across the experimental combinations, thus raising questions about their effectiveness as indicators of graft incompatibility in chestnut.

In conclusion, studying this issue remains challenging for various reasons. Current scientific findings support an integrated multidisciplinary approach rather than one relying on single methods. The combined study of chemical and physiological parameters has provided deeper insights into graft incompatibility. Further research will, therefore, have to focus on validating these findings by enlarging the number of genotypes tested, broadening the range of phenolic markers considered, and investigating potential non-linear correlations. In addition, exploring histological aspects, such as phenol accumulation in tissues through staining techniques, could provide deeper insights into the dynamics of these secondary metabolites. The ultimate goal is to establish a practical and routine diagnostic tool for graft incompatibility, which would aid breeders and researchers in developing new, resilient rootstocks essential for advancing chestnut cultivation.

## Supplementary Information

Below is the link to the electronic supplementary material.

Supplementary file1 (DOCX 20 KB)

Supplementary file2 (DOCX 20 KB)

## Data Availability

The data supporting the findings of this study are available from the corresponding author, Giovanni Gamba, upon request.

## Abbreviations

BBxCA07: Bouche de Bétizac × Marsol CA07
CAL: Callusing
EVC: End vegetative cycle
MSxCA07: Marrone Val Susa × Marsol CA07
MSxCren: Marrone Val Susa × *C. crenata*
MSxMoll: Marrone Val Susa × *C. mollissima*
TPC: Total phenolic content
