# Supplementary material for: Insights into chestnut (Castanea spp.) graft incompatibility through the monitoring of chemical and physiological parameters
Source: Planta. 2025 Feb 14;261(3):60. doi: 10.1007/s00425-025-04639-8 (PMC11828799; doi:10.1007/s00425-025-04639-8)
Supplement: Supplementary file 1 — Supplementary file1 (DOCX 20 KB) [file 425_2025_4639_MOESM1_ESM.docx]

|  |  |  | **Benzoic acids** | | **Catechins** | | **Tannins** | |
| --- | --- | --- | --- | --- | --- | --- | --- | --- |
| **Combination** | **section** | **tissues** | **ellagic acid** | **gallic acid** | **catechin** | **epicatechin** | **castalagin** | **vescalagin** |
|  |  |  | *(mg/100 gFW)* | *(mg/100 gFW)* | *(mg/100 gFW)* | *(mg/100 gFW)* | *(mg/100 gFW)* | *(mg/100 gFW)* |
| **MSxCA07** | scion | external | <0.061 | <0.044 | <0.234 | 832.983 | 130.404 | 36.907 |
|  |  | internal | <0.061 | <0.044 | <0.234 | 12.929 | 43.321 | <0.060 |
|  | graft | external | <0.204 | <0.044 | <0.234 | 20.125 | 121.497 | 5.661 |
|  |  | internal | <0.061 | <0.044 | <0.234 | 16.377 | 34.149 | 7.387 |
|  | rootstock | external | 6.644 | <0.044 | <0.234 | 428.230 | 1466.966 | 38.007 |
|  |  | internal | <0.061 | <0.044 | <0.234 | 11.752 | 17.802 | 6.458 |
| **BBxCA07** | scion | external | 4.205 | <0.044 | 404.878 | 465.105 | 4155.483 | 83.247 |
|  |  | internal | <0.061 | <0.145 | <0.234 | 17.465 | 36.914 | 6.710 |
|  | graft | external | <0.204 | <0.044 | <0.234 | 290.119 | 2247.703 | 36.895 |
|  |  | internal | <0.061 | <0.044 | <0.234 | 16.183 | 43.111 | 5.968 |
|  | rootstock | external | 8.294 | 36.122 | 264.755 | 684.021 | 1816.065 | 42.657 |
|  |  | internal | <0.061 | <0.044 | <0.234 | 11.301 | 23.712 | 6.329 |
| **MSxCren** | scion | external | <0.061 | <0.044 | <0.234 | 229.692 | 2632.951 | 56.735 |
|  |  | internal | <0.061 | <0.044 | <0.234 | 23.502 | 65.399 | 6.132 |
|  | graft | external | <0.204 | <0.044 | <0.234 | 173.219 | 1248.218 | 44.204 |
|  |  | internal | <0.204 | <0.044 | <0.234 | 19.776 | 33.681 | 9.235 |
|  | rootstock | external | <0.204 | <0.044 | 439.325 | 876.135 | 1142.014 | 33.105 |
|  |  | internal | <0.061 | <0.044 | <0.234 | 12.807 | 30.231 | 4.884 |
| **MSxMoll** | scion | external | 3.669 | <0.044 | <0.234 | 384.554 | 2831.728 | 125.705 |
|  |  | internal | <0.061 | <0.044 | <0.234 | 20.713 | 55.989 | 4.348 |
|  | graft | external | 1.302 | 23.730 | <0.234 | 103.039 | 933.707 | 28.853 |
|  |  | internal | <0.061 | <0.044 | <0.234 | 12.065 | 20.560 | 11.719 |
|  | rootstock | external | 15.527 | <0.044 | <0.234 | 284.073 | 5995.223 | 198.682 |
|  |  | internal | <0.061 | <0.044 | 2.666 | 9.016 | 30.645 | 14.402 |

**Table S1** Polyphenolic fingerprint of the tissues at the graft union. The results are reported as mg of the bioactive compound per 100 g fresh weight (FW). Values refer to the callusing (CAL) stage. The mean value is given for each sample (n = 3)
