# Supplementary material for: Insights into chestnut (Castanea spp.) graft incompatibility through the monitoring of chemical and physiological parameters
Source: Planta. 2025 Feb 14;261(3):60. doi: 10.1007/s00425-025-04639-8 (PMC11828799; doi:10.1007/s00425-025-04639-8)
Supplement: Supplementary file 2 — Supplementary file2 (DOCX 20 KB) [file 425_2025_4639_MOESM2_ESM.docx]

|  |  |  | **Benzoic acids** | | **Catechins** | | **Tannins** | |
| --- | --- | --- | --- | --- | --- | --- | --- | --- |
| **Combination** | **section** | **tissues** | **ellagic acid** | **gallic acid** | **catechin** | **epicatechin** | **castalagin** | **vescalagin** |
|  |  |  | *(mg/100 gFW)* | *(mg/100 gFW)* | *(mg/100 gFW)* | *(mg/100 gFW)* | *(mg/100 gFW)* | *(mg/100 gFW)* |
| **MSxCA07** | scion | external | <0.204 | 141.503 | 72.867 | 595.235 | 1582.931 | 75.551 |
|  |  | internal | <0.061 | <0.044 | <0.234 | 144.136 | 328.988 | 10.858 |
|  | graft | external | <0.204 | 105.545 | 187.148 | 251.340 | 1653.207 | 67.687 |
|  |  | internal | <0.204 | 54.799 | 58.953 | 162.792 | 439.425 | 10.620 |
|  | rootstock | external | 12.067 | <0.044 | 596.696 | 399.936 | 1749.566 | 42.345 |
|  |  | internal | <0.204 | <0.044 | 54.470 | 112.454 | 160.818 | 6.186 |
| **BBxCA07** | scion | external | 14.739 | <0.044 | 462.207 | 734.861 | 2430.649 | 67.186 |
|  |  | internal | <0.061 | 9.124 | <0.234 | 41.566 | 40.626 | 3.126 |
|  | graft | external | 11.104 | <0.044 | 545.037 | 394.020 | 2274.309 | 66.442 |
|  |  | internal | <0.204 | 8.952 | <0.234 | 45.641 | 51.393 | 4.811 |
|  | rootstock | external | 21.120 | <0.044 | 902.965 | 376.647 | 1208.680 | 39.378 |
|  |  | internal | <0.204 | <0.145 | 41.202 | 22.627 | 21.629 | 3.679 |
| **MSxCren** | scion | external | <0.204 | 95.408 | 85.138 | 620.596 | 2937.280 | 120.414 |
|  |  | internal | <0.204 | <0.044 | 23.216 | 191.319 | 480.973 | 23.908 |
|  | graft | external | <0.204 | <0.044 | 267.789 | 473.583 | 2808.724 | 73.209 |
|  |  | internal | <0.204 | 100.029 | 98.756 | 352.399 | 1716.329 | 52.088 |
|  | rootstock | external | <0.061 | <0.044 | 261.749 | 531.324 | 1407.888 | 72.189 |
|  |  | internal | <0.061 | <0.044 | 29.685 | 39.428 | 33.366 | 7.026 |
| **MSxMoll** | scion | external | <0.204 | <0.044 | 111.614 | 830.570 | 3204.463 | 147.823 |
|  |  | internal | <0.204 | 11.131 | 12.575 | 225.653 | 292.974 | 26.257 |
|  | graft | external | 51.644 | <0.044 | <0.234 | 296.930 | 2791.214 | 79.848 |
|  |  | internal | <0.204 | <0.044 | <0.234 | 6.838 | 8.080 | 9.834 |
|  | rootstock | external | 9.152 | <0.044 | <0.234 | 104.290 | 2218.772 | 41.888 |
|  |  | internal | <0.204 | <0.044 | <0.234 | 16.614 | 49.236 | 3.992 |

**Table S2** Polyphenolic fingerprint of the tissues at the graft union. The results are reported as mg of the bioactive compound per 100 g fresh weight (FW). Values refer to the end of the vegetative cycle (EVC) stage. The mean value is given for each sample (n = 3)
